# The MOTHER (Major Obstetric Trauma or Haemorrhage EmeRgency) guideline: development of an algorithm for emergency department management of trauma in pregnancy

**DOI:** 10.1186/1757-7241-23-S2-A5

**Published:** 2015-09-11

**Authors:** Nathan Borgeaud, Sumitra Lahiri, Anna Dobbie

**Affiliations:** 1 Department of Anaesthetics, Royal London Hospital, London, UK; 2 Department of Emergency Medicine, Royal London Hospital, London, UK

## Introduction

Trauma is now the leading cause of non-obstetric death in pregnancy. Emergency department (ED) management of trauma in pregnancy may be complicated by physiological compensation for concealed haemorrhage, reduced accuracy of diagnostic examination, a need to coordinate multiple teams, worries about imaging radiation and difficult decisions regarding emergent fetal delivery. Time constraints and emotive circumstances contribute to the challenge. We aimed to develop an evidence-based guideline for early management of trauma or significant haemorrhage concern in pregnant patients presenting to the ED.

## Methods

We conducted a non-systematic literature review of publications pertaining to key components of management of trauma in pregnancy. Based on this, as well as experience from within a major trauma centre in London, an algorithm was developed. A multidisciplinary team involving emergency, obstetric, anaesthetic, radiology and neonatal departments contributed. Existing local guidelines were reviewed for incorporation, as well as nationally published recommendations taught within the MOET (Managing Obstetric Emergencies and Trauma) course.

## Results

The MOTHER algorithm was created to include pregnant females of ≥20 weeks gestation. It was reproduced in a clear visual format, identifying key resuscitation principles and adopting a stepwise systematic approach. Subsections provided guidance on peri-mortem caesarean as well as management of patients with minimal trauma. Specialty-specific appendices provided individualised team guidance. A decision making tool was developed to aid surgical decisions in abdominal trauma.

## Discussion

MOTHER is a simple evidence-based algorithm that aids decisions in the ED, enhances understanding of important physiological principles, incorporates existing resuscitation recommendations and promotes collaboration between teams. As a result MOTHER may reduce adverse events and improve both maternal and fetal outcome. We believe the evidence reviewed is sufficient to recommend adoption of MOTHER at a national level.

